# Prospective External Validation of the HFA-ICOS Baseline Cardiovascular Risk Classification in Women with Breast Cancer Receiving Anthracycline-Containing Therapy in a Central Asian Cohort

**DOI:** 10.3390/diagnostics16203379

**Published:** 2026-10-17

**Authors:** Aiganym Amanova, Bekbolat Zholdin, Saule Balmagambetova, Iliada Talipova, Gulnara Kurmanalina, Saule Madinova, Mira Baspayeva, Kulparshan Kubenova, Gulmira Sultanbekova, Maria Poltavskaya, Zhenisgul Tlegenova

**Affiliations:** 1 Department of Internal Diseases-2, West Kazakhstan Marat Ospanov Medical University, Aktobe 030019, Kazakhstan; amanova.a@zkmu.kz (A.A.); bekbolat.zholdin@gmail.com (B.Z.); iliada61@mail.ru (I.T.); gulnara.kurmanalina@mail.ru (G.K.); ainika-90@list.ru (G.S.); 2 Department of Oncology, West Kazakhstan Marat Ospanov Medical University, Aktobe 030019, Kazakhstan; 3 Chemotherapy Division, University Medical Center, West Kazakhstan Marat Ospanov Medical University, Aktobe 030019, Kazakhstan; saule.madi.85@mail.ru (S.M.); miranelle@mail.ru (M.B.); 4 Clinical Laboratory, University Medical Center, West Kazakhstan Marat Ospanov Medical University, Aktobe 030017, Kazakhstan; kkubenova@mail.ru; 5 Department of Cardiology, Functional and Ultrasound Diagnostics, Sechenov First Moscow State Medical University, Moscow 119991, Russia; poltavskaya_m_g@staff.sechenov.ru

**Keywords:** HFA-ICOS, cardio-oncology, cancer therapy-related cardiac dysfunction, anthracycline, breast cancer, external validation, cardiotoxicity, Central Asia

## Abstract

**Background/Objectives:** The HFA-ICOS baseline cardiovascular risk classification was developed to standardize pretreatment risk stratification before potentially cardiotoxic cancer therapy, but its external performance across diverse populations remains incompletely characterized. We prospectively evaluated its performance in women with breast cancer receiving anthracycline-containing therapy in Central Asia. **Methods:** This prospective single-center external validation study used data from the PREDICATE cohort (ISRCTN12628444). Participants were classified at baseline as being at low, moderate, high, or very high cardiovascular risk according to the HFA-ICOS classification before treatment and outcome ascertainment; the high- and very-high-risk categories were pooled for analysis because of small numbers. Participants underwent serial cardiovascular surveillance, including echocardiographic assessment of left ventricular ejection fraction and global longitudinal strain, together with serial cardiac biomarker measurements. The primary outcome was cancer therapy-related cardiac dysfunction (CTRCD), defined according to the 2022 ESC cardio-oncology criteria. **Results:** The analysis included 108 participants. CTRCD occurred in 43 of 108 participants (39.8%). The observed proportion of participants who developed CTRCD increased from 30.0% in the low-risk group to 47.5% in the moderate-risk group and 75.0% in the high/very-high-risk group (*p* = 0.026). Kaplan–Meier analysis demonstrated significant separation of CTRCD-free survival across HFA-ICOS risk categories (log-rank χ^2^ = 10.12; *p* = 0.006). Compared with the low-risk group, the hazard of CTRCD was higher in the moderate-risk group (hazard ratio [HR], 2.16; 95% confidence interval [CI], 1.13–4.13) and high/very-high-risk group (HR, 3.59; 95% CI, 1.42–9.11). Discrimination was modest (area under the curve, 0.630; 95% CI, 0.530–0.724). **Conclusions:** The HFA-ICOS classification demonstrated a clear ordinal gradient in CTRCD risk and significant time-to-event separation but showed limited discrimination at the individual-patient level. It may guide initial risk stratification but should be complemented by longitudinal cardiovascular surveillance rather than used as a stand-alone predictor.

## 1. Introduction

Anthracyclines remain a cornerstone of neoadjuvant and adjuvant therapy for breast cancer (BC) [1,2], but their use is associated with a risk of cancer therapy-related cardiac dysfunction (CTRCD), which may develop during treatment or become apparent during subsequent follow-up [3,4,5]. Early myocardial injury is often clinically silent but may progress, compromise anticancer treatment, and adversely affect long-term cardiovascular outcomes [6]. Pretreatment identification of patients at increased cardiovascular risk is therefore important for guiding risk-adapted surveillance and optimizing modifiable cardiovascular risk factors [7].

The Heart Failure Association (HFA) of the European Society of Cardiology (ESC), in collaboration with the International Cardio-Oncology Society (ICOS), developed the HFA-ICOS baseline cardiovascular risk assessment tool, which classifies patients as being at low, moderate, high, or very high risk before potentially cardiotoxic therapy [8]. The tool was subsequently incorporated into the 2022 ESC Guidelines on Cardio-Oncology [9]. However, its external performance has varied across study populations. In a recent meta-analysis, most validation studies were conducted in patients receiving HER2-targeted therapy, in whom discrimination was modest (pooled C-statistic 0.60; 95% CI 0.52–0.68) and risk was systematically underestimated [10]. In anthracycline-treated cohorts, CTRCD incidence generally increased across HFA-ICOS risk categories, whereas discrimination ranged from comparatively strong in the multicenter CARDIOTOX Registry [11] to more modest in the Japanese AIC Registry [12] and several smaller BC cohorts [13,14,15].

The performance of a risk classification may depend partly on the case mix and clinical setting of the population in which it is applied [16]. The generalizability of existing HFA-ICOS findings to Central Asian populations remains uncertain because both the distribution of baseline risk factors and the conditions under which CTRCD is detected may differ from those in previously studied cohorts [17,18,19]. Central Asia has a high burden of cardiovascular disease, with a substantial contribution from cardiometabolic risk factors, particularly arterial hypertension and diabetes mellitus, both of which are incorporated into the HFA-ICOS baseline risk assessment [18,20]. In Kazakhstan, BC is diagnosed at a comparatively young mean age of approximately 56 years [21], which may further influence baseline risk classification because age is also an explicit component of the HFA-ICOS tool. At the same time, differences in cardio-oncology services and surveillance pathways may affect how early and how consistently CTRCD is identified [17]. Together, these differences in baseline risk profile and outcome ascertainment support the need for prospective validation of HFA-ICOS in this setting rather than assuming direct transferability from previously studied populations.

Accordingly, we evaluated the external performance of prospectively assigned HFA-ICOS baseline cardiovascular risk categories in women with BC receiving anthracycline-containing therapy in Kazakhstan. We assessed the ordinal gradient in CTRCD occurrence, differences in time to first CTRCD across baseline risk categories, and overall discrimination.

## 2. Materials and Methods

### 2.1. Study Design and Population

The present study was an external validation analysis of the HFA-ICOS baseline cardiovascular risk classification using prospectively collected longitudinal data from the Prospective Risk Evaluation for the Development of Anthracycline-Induced Cardiotoxicity (PREDICATE) cohort. PREDICATE is a prospective, single-center observational cohort study conducted at the Medical Center of Marat Ospanov West Kazakhstan Medical University, Aktobe, Kazakhstan. The design and methodology of the parent PREDICATE study have been published previously [22], and the study was registered with the ISRCTN registry (ISRCTN12628444).

Between September 2021 and August 2022, consecutive women aged ≥18 years with histologically confirmed BC who were candidates for neoadjuvant or adjuvant treatment with anthracyclines and/or HER2-targeted therapy were enrolled; full eligibility criteria have been described previously [22]. Patients with severe pre-existing left ventricular systolic dysfunction (LVEF < 40%), decompensated heart failure, progression of clinically significant cardiovascular disease, or decompensation of another severe comorbid condition were not eligible for enrollment.

For the present external validation, the analysis was restricted to participants who received anthracycline-containing chemotherapy, with or without HER2-targeted therapy. Participants who received trastuzumab without anthracycline exposure were therefore excluded.

The study was conducted in accordance with the principles of the Declaration of Helsinki and was approved by the local ethics committee of the West Kazakhstan Marat Ospanov Medical University (Ref. No. 7, 9 September 2020; Ref. No. 11, 24 November 2023) [23]. All participants provided written informed consent before enrollment.

### 2.2. Baseline Cardiovascular Assessment and HFA-ICOS Risk Classification

Before initiation of anticancer therapy, all participants underwent a standardized baseline cardio-oncology assessment, including medical history, physical examination, anthropometric measurements, evaluation of pre-existing cardiovascular disease and conventional cardiovascular risk factors, laboratory testing, 12-lead electrocardiography, and comprehensive transthoracic echocardiography [24]. Cancer characteristics and the planned anticancer treatment strategy were prospectively recorded at the baseline visit, before initiation of anticancer therapy. Baseline cardiovascular risk was classified using the HFA-ICOS risk assessment tool applicable to patients scheduled to receive anthracycline-containing chemotherapy [8]. Its implementation in the PREDICATE cohort has been described previously [19].

The HFA-ICOS assessment incorporated pre-existing cardiovascular disease, baseline cardiac imaging findings, cardiac biomarkers, age, cardiovascular risk factors, and previous exposure to potentially cardiotoxic cancer therapies and assigned participants to one of four predefined baseline cardiovascular risk categories—low, moderate, high, or very high—according to the original HFA-ICOS framework [9]. The individual HFA-ICOS components applied in the PREDICATE cohort are detailed in Supplementary Table S1.

Baseline HFA-ICOS categories were assigned prospectively, before the first treatment cycle and before any study outcome, using only pretreatment information. The original classification was applied without recalibration, modification of risk factors, weights or thresholds, or addition of predictors, and participants were not reclassified on the basis of any post-baseline clinical, imaging, or biomarker findings.

### 2.3. Anticancer Treatment and Anthracycline Exposure

Anticancer treatment was prescribed by the treating oncology team according to tumor characteristics and contemporary clinical protocols [25].

Anthracycline-containing treatment regimens were classified based on oncology treatment records as doxorubicin–cyclophosphamide (AC), AC followed by a taxane (AC-T), AC followed by taxane- and trastuzumab-containing HER2-targeted therapy (AC-TH), and docetaxel–doxorubicin–cyclophosphamide (TAC). Anthracycline exposure—including the agent administered, treatment regimen, number of cycles, and cumulative dose—was prospectively recorded from oncology treatment records. Cumulative anthracycline exposure was expressed as the total doxorubicin dose (mg/m^2^) administered throughout treatment. Information on HER2-targeted therapy, thoracic radiotherapy, treatment modifications or interruptions, and cardiovascular medications prescribed before or during anticancer treatment was recorded prospectively. Detailed treatment characteristics are provided in Table 1 and Supplementary Table S2.

### 2.4. Cardiovascular Risk Factor Management and Cardioprotective Therapy

Baseline cardiovascular risk factors were assessed and, when clinically indicated, managed before the first cycle of anthracycline therapy. In patients with arterial hypertension, antihypertensive therapy was initiated or optimized, with angiotensin-converting enzyme inhibitors (ACEIs) or angiotensin receptor blockers (ARBs) used as preferred agents in the absence of contraindications [26]. Statins were prescribed according to cardiovascular risk and low-density lipoprotein cholesterol levels [27]. In patients with diabetes mellitus, glucose-lowering therapy was optimized, including sodium–glucose cotransporter-2 (SGLT2) inhibitors when clinically indicated [28]. Beta-blockers were prescribed for patients with chronic coronary syndrome, heart rate control in atrial fibrillation or other tachyarrhythmias, and hypertension when clinically appropriate [3]. Pre-existing cardiovascular therapy was continued and optimized when necessary.

Dexrazoxane and liposomal anthracycline formulations were not used because they were not available at the study center during the study period.

During follow-up, cardiovascular therapy was initiated or intensified when new cardiovascular indications emerged or CTRCD developed. In patients with mild asymptomatic CTRCD, ACEIs/ARBs and/or beta-blockers were initiated or optimized when clinically appropriate, while anthracycline-containing therapy was continued with closer cardiovascular monitoring. In patients with moderate asymptomatic CTRCD, guideline-directed cardioprotective therapy, including ACEIs/ARBs and/or beta-blockers when clinically appropriate and in the absence of contraindications, was initiated or optimized. Temporary interruption of anthracycline-containing therapy was considered in accordance with the 2022 ESC Guidelines on Cardio-Oncology [9], with subsequent decisions regarding continuation or resumption made jointly by cardiologists and oncologists after reassessment of LV function, clinical status, and oncological indications.

For the analysis, cardioprotective therapy was classified according to its timing relative to anthracycline treatment as baseline therapy (ongoing or initiated before the first anthracycline cycle) or post-baseline therapy (newly initiated after the start of anthracycline therapy).

### 2.5. Echocardiographic Assessment

Comprehensive transthoracic echocardiography was performed before initiation of anticancer therapy and repeated prospectively during treatment and post-treatment follow-up, including conventional two-dimensional imaging and speckle-tracking assessment of LV global longitudinal strain [29]. All echocardiographic examinations were performed using a Vivid E9 ultrasound system (GE HealthCare, Horten, Norway). LV end-diastolic and end-systolic volumes were measured using the biplane modified Simpson method, and LVEF was calculated accordingly [30]. LV GLS was assessed by two-dimensional speckle-tracking echocardiography from standard apical four-chamber, two-chamber, and three-chamber views [31]. Endocardial borders were automatically detected and visually reviewed, with manual adjustment of the region of interest when required to ensure adequate myocardial tracking. GLS was calculated from adequately tracked LV myocardial segments across the three apical views according to established consensus definitions [31,32]. The relative change in GLS from baseline was calculated for CTRCD ascertainment [9,32]. When a relative GLS decline > 15% from baseline was identified, the measurement was repeated at a confirmatory examination within 1 week, and a GLS-based CTRCD event was recorded only if the decline was confirmed. To minimize inter-vendor and inter-software variability, all serial examinations for an individual participant were acquired using the same ultrasound system and analyzed with the same software version, by the same observer whenever feasible. All echocardiographic examinations were performed and analyzed by two certified cardiologists with 3 and 10 years of experience in echocardiography, respectively, working within the same echocardiography laboratory. Reproducibility of echocardiographic measurements was assessed in a subgroup of 16 randomly selected participants. Intra-observer and inter-observer agreement for LVEF and LV GLS were quantified using the intraclass correlation coefficient (ICC) with corresponding 95% confidence intervals, and measurement precision was additionally expressed as the coefficient of variation (CoV) [33]. Systematic bias and limits of agreement were visualized using Bland–Altman plots (Supplementary Figure S1). Researchers were blinded to participants’ baseline HFA-ICOS risk category at all follow-up assessments; however, results of participants’ previous echocardiographic examinations were available at the time of subsequent analyses. Examinations in which reliable GLS assessment could not be achieved because of inadequate image quality or myocardial tracking were considered technically unsuitable for GLS analysis and treated as missing for the GLS component of CTRCD ascertainment, while the corresponding LVEF measurement, when technically adequate, was retained for analysis.

### 2.6. Cardiac Biomarkers

Cardiac troponin I (cTnI) and B-type natriuretic peptide (BNP) were measured at baseline, before initiation of anticancer therapy, and serially during prospective cardiovascular surveillance. Blood sampling during active anticancer treatment was performed before administration of the corresponding treatment cycle whenever feasible.

cTnI and BNP concentrations were measured at the clinical laboratory of the study center using immunofluorescence-based rapid quantitative assays (Finecare™ rapid quantitative tests; Guangzhou Wondfo Biotech Co., Ltd., Guangzhou, China) in accordance with the manufacturer’s instructions and institutional laboratory procedures. According to the assay-specific reference thresholds applied during the study, cTnI values >0.30 ng/mL and BNP values >100 pg/mL were considered elevated (the reported thresholds were derived from the manufacturers’ analyzer manuals).

Baseline cTnI and BNP measurements obtained before initiation of anticancer therapy were used for HFA-ICOS cardiovascular risk classification. Serial post-baseline cTnI and BNP measurements were used for longitudinal biomarker assessment and, where applicable, for ascertainment of the biomarker component of CTRCD.

### 2.7. CTRCD Definition and Outcome Ascertainment

The primary outcome of the present external validation study was time to first CTRCD from the initiation of anthracycline-containing therapy. Outcome ascertainment encompassed the entire prospective observation period, including both active anticancer treatment and post-treatment follow-up.

CTRCD was classified according to the 2022 ESC Guidelines on Cardio-Oncology, incorporating changes in LVEF, LV GLS, cardiac biomarkers, and clinical heart failure status [9]. Asymptomatic CTRCD was classified as mild, moderate, or severe. Mild asymptomatic CTRCD was defined as preserved LVEF ≥ 50% accompanied by a new relative decline in LV GLS greater than 15% from baseline and/or a new rise in cardiac biomarkers consistent with myocardial injury [9,34,35]. Moderate asymptomatic CTRCD was defined as either a new LVEF reduction by ≥10 percentage points to an LVEF of 40–49%, or a new LVEF reduction by <10 percentage points to an LVEF of 40–49% accompanied by either a new relative decline in LV GLS > 15% from baseline or a new rise in cardiac biomarkers [9]. Severe asymptomatic CTRCD was defined as a new reduction in LVEF to <40% [9]. Symptomatic CTRCD was diagnosed in patients who developed clinical heart failure according to the 2022 ESC criteria [9].

This differs from the composite endpoint used in an earlier interim report from this cohort [36], which combined echocardiographic decline, treatment discontinuation, and cardiovascular death; consequently, event counts and outcome ascertainment in the present analysis are not directly comparable to that earlier report. The event date was defined as the date of the first scheduled or unscheduled cardiovascular assessment at which the CTRCD criteria were documented; only the first qualifying event was considered for the primary outcome.

### 2.8. Follow-Up and Cardiovascular Surveillance

Prospective cardiovascular surveillance was conducted according to the PREDICATE study protocol and extended from baseline assessment before initiation of anthracycline-containing therapy through active anticancer treatment and subsequent post-treatment follow-up. During active treatment, protocol-scheduled cardiovascular assessments were performed at approximately 3-month intervals and at treatment completion. Because the duration of anticancer treatment varied among participants, the number of assessments during active treatment varied accordingly, and the treatment-completion assessment could coincide with a scheduled 3-month assessment.

After completion of anticancer treatment, participants continued prospective follow-up within the PREDICATE cohort, with scheduled cardiovascular assessments performed at approximately 12-month intervals. In addition to protocol-scheduled visits, clinically indicated unscheduled cardiovascular evaluations could be performed at any time during active treatment or post-treatment follow-up when new cardiovascular symptoms, deterioration in clinical status, or suspected CTRCD warranted additional assessment. Scheduled and clinically indicated cardiovascular assessments included clinical evaluation, transthoracic echocardiography with measurement of LVEF and LV GLS, and cardiac biomarker assessment [37,38]. During active anticancer treatment, cardiovascular investigations were performed before administration of the corresponding treatment cycle whenever feasible. Both scheduled and unscheduled assessments were considered for CTRCD ascertainment using the same predefined criteria. For the present HFA-ICOS external validation analysis, all PREDICATE follow-up data up to January 2025 were included.

### 2.9. Statistical Analysis

Continuous variables were assessed for normality using the Shapiro–Wilk test and visual inspection of distributions and are presented as mean ± standard deviation or median [interquartile range], as appropriate. Categorical variables are presented as counts and percentages.

No separate a priori sample-size calculation was performed for the present external validation analysis; the sample size of the parent PREDICATE cohort was determined a priori as described in the published study protocol [22] (target N = 120, to account for anticipated losses during follow-up from an original calculated requirement of 100).

Baseline characteristics were summarized overall and by HFA-ICOS risk category and compared using one-way analysis of variance or the Kruskal–Wallis test for continuous variables and the χ^2^ test or Fisher’s exact test for categorical variables, as appropriate. Because several baseline characteristics are components of the HFA-ICOS classification, corresponding between-group *p* values were interpreted descriptively. Because of the sparse representation of the high- and very-high-risk categories, these two categories were pooled into a single high/very-high-risk stratum for inferential analyses. This decision was made after the distribution of participants across baseline HFA-ICOS categories had been established but before CTRCD outcomes were examined by risk category. Pooling did not alter individual HFA-ICOS assignments.

External validation assessed observed risk stratification, discrimination, and time-to-event separation [39,40]. Observed CTRCD proportions with 95% confidence intervals (CIs) were calculated for the low-, moderate-, and pooled high/very-high-risk strata. Discrimination was evaluated by receiver operating characteristic analysis, with HFA-ICOS category treated as an ordinal predictor; the area under the curve (AUC) and 95% CI were estimated using 5000 bootstrap resamples. Because HFA-ICOS is a multi-category risk stratification instrument rather than a binary diagnostic test, dichotomized classification metrics were not considered primary measures of its performance.

Because HFA-ICOS provides categorical risk strata rather than individualized probabilities of CTRCD, calibration-in-the-large and calibration slope were not estimated. Observed CTRCD proportions across the predefined categories were instead used to characterize the risk gradient. No recalibration, re-estimation of predictor weights, modification of risk thresholds, or addition of predictors was performed.

Time to first CTRCD was calculated from the initiation of anthracycline-containing therapy to the first qualifying event. Participants who remained free of CTRCD were censored at the date of their last available cardiovascular assessment before the January 2025 data cutoff.

CTRCD-free survival was estimated using the Kaplan–Meier method and compared across HFA-ICOS categories using the log-rank test. Cox proportional-hazards regression was used to estimate hazard ratios (HRs) and 95% CIs, with low risk as the reference category. The proportional-hazards assumption was assessed using Schoenfeld residuals.

To assess the potential influence of baseline cardiovascular/cardioprotective medication use and treatment-related exposures, an additional Cox proportional-hazards model was performed as an exploratory sensitivity analysis. The model adjusted for baseline HFA-ICOS risk category, baseline use of ACEIs/ARBs and/or beta-blockers, cumulative doxorubicin dose (per 50 mg/m^2^ increase), and left-sided thoracic radiotherapy.

Baseline HFA-ICOS classification, baseline LVEF, LV GLS, cTnI and BNP, primary CTRCD outcome status, and time-to-event or censoring information were available for all 108 participants; therefore, the primary validation analyses included the entire cohort without imputation. Missing serial post-baseline echocardiographic and biomarker measurements were not imputed, and analyses requiring these measurements used available observations. Technically inadequate LV GLS measurements were treated as missing for GLS-based analyses while other available measurements from the same assessment were retained.

All statistical tests were two-sided, and *p* < 0.05 was considered statistically significant. Analyses were performed using IBM SPSS Statistics version 26.0 (IBM Corp., Armonk, NY, USA) and R 4.3.1 (R Foundation for Statistical Computing, Vienna, Austria).

Analyses other than the primary validation analyses should be interpreted as exploratory.

## 3. Results

### 3.1. Study Population and Baseline Characteristics

Between September 2021 and August 2022, 128 consecutive women with BC were enrolled in the prospective PREDICATE cohort. Of these, 120 participants completed the original longitudinal study protocol, as previously reported [41]. Eight participants did not complete the protocol because of oncological death due to disease progression (*n* = 4), early withdrawal or loss to follow-up (*n* = 3), or discontinuation of anticancer therapy after Visit 2 (*n* = 1). The derivation of the external validation cohort is shown in Figure 1.

The present analysis included 108 women with BC exposed to anthracycline-containing therapy. According to the prospectively assigned baseline HFA-ICOS classification, 60 participants (55.6%) were classified as low risk, 40 (37.0%) as moderate risk, and 8 (7.4%) as high/very-high risk (Supplementary Table S1).

Baseline demographic, cardiovascular, oncological, and treatment characteristics of the cohort are summarized in Table 1. Participants in higher HFA-ICOS categories were older and had a greater burden of cardiovascular comorbidity. Baseline echocardiographic parameters also differed across risk strata. Median LVEF decreased across HFA-ICOS risk categories, from 61.0% [IQR 58.0–63.0] in the low-risk group to 57.0% [53.0–59.0] in the high/very-high-risk group (*p* < 0.001). Median LV GLS showed a similar pattern, ranging from −18.6% [−19.7 to −17.8] in the low-risk group to −16.7% [−19.4 to −15.5] in the high/very-high-risk group (*p* = 0.002). Corresponding cardiac biomarker values are presented in Table 1. Because several of these characteristics are components of the HFA-ICOS classification itself (Supplementary Table S1), between-group comparisons should be interpreted descriptively rather than as independent associations with HFA-ICOS risk.

All participants included in the validation cohort received doxorubicin-containing anthracycline therapy. Of the 108 participants, 98 (90.7%) received anthracycline-containing chemotherapy without trastuzumab, whereas 10 (9.3%) received anthracycline-containing therapy followed by trastuzumab-based HER2-targeted treatment. Median cumulative doxorubicin exposure was 237.6 mg/m^2^ [IQR 192.2–315.9], with an observed range of 51.4–583.7 mg/m^2^. Radiotherapy was recorded in 95 participants (88.0%). Detailed anticancer treatment exposure, including chemotherapy regimen and radiotherapy laterality, as well as post-baseline cardiovascular/cardioprotective therapy, is provided in Supplementary Table S2.

Baseline cardiovascular medication use differed across HFA-ICOS risk categories. ACEIs/ARBs were used in 56 participants (51.9%), including 13/60 (21.7%) in the low-risk group, 35/40 (87.5%) in the moderate-risk group, and 8/8 (100.0%) in the high/very-high-risk group. Beta-blockers were used in 2 participants (1.9%), both in the high/very-high-risk group. Statins were used in 79 participants (73.1%), with similar proportions across the low-, moderate-, and high/very-high-risk groups (75.0%, 70.0%, and 75.0%, respectively). No participants were receiving an SGLT2 inhibitor at baseline (Table 1).

In the randomly selected subset of 16 participants, the intra- and inter-observer ICCs for LV GLS were 0.94 (95% CI 0.86–0.97) and 0.90 (95% CI 0.83–0.96), with corresponding coefficients of variation (CoV) of 1.88% and 2.12%, respectively. For LVEF, the intra- and inter-observer ICCs were 0.91 (95% CI 0.82–0.94) and 0.89 (95% CI 0.78–0.94), with CoVs of 3.70% and 4.86%. Bland–Altman analysis demonstrated the corresponding mean biases and 95% limits of agreement for both LVEF and LV GLS measurements (Supplementary Figure S1). The timing, ascertainment, and severity of first CTRCD events during active treatment and post-treatment follow-up are reported separately in Supplementary Table S3.

### 3.2. Occurrence and Timing of CTRCD Across HFA-ICOS Risk Categories

During the entire prospective observation period, encompassing active anticancer treatment and post-treatment follow-up, 43 of 108 participants (39.8%) experienced at least one CTRCD event. The median time from initiation of anthracycline-containing therapy to the first documented CTRCD was 361 days (IQR 264–442 days). The observed proportion of participants developing CTRCD increased progressively across baseline HFA-ICOS risk categories, from 30.0% (18/60) in the low-risk group to 47.5% (19/40) in the moderate-risk group and 75.0% (6/8) in the high/very-high-risk group (overall *p* = 0.026, Fisher–Freeman–Halton exact test). Corresponding 95% confidence intervals are presented in Table 2.

First CTRCD events occurred both during active anthracycline-containing therapy and during post-treatment follow-up. Of the 43 first CTRCD events, 34 (79.1%) were documented during active anthracycline-containing treatment or at the treatment-completion assessment, whereas 9 (20.9%) were first documented during post-treatment follow-up. Most first CTRCD events were mild and asymptomatic (37/43, 86.0%), while 6 (14.0%) were classified as moderate asymptomatic CTRCD; no severe asymptomatic or symptomatic first CTRCD events were recorded. All 43 primary events were supported by echocardiographic criteria. Detailed information on the timing, severity, and basis of ascertainment of first CTRCD events is provided in Supplementary Table S3.

### 3.3. Time to First CTRCD According to Baseline HFA-ICOS Risk

The median duration of prospective follow-up from initiation of anthracycline-containing therapy was 1015 days (IQR 728–1126; range 70–1224). Kaplan–Meier analysis demonstrated clear separation of CTRCD-free survival curves across baseline HFA-ICOS risk categories (Figure 2). Participants classified as low risk had the highest probability of remaining free from CTRCD, whereas progressively lower CTRCD-free survival was observed in the moderate- and high/very-high-risk groups. The overall difference between the curves was statistically significant (log-rank χ^2^ = 10.12, *p* = 0.006).

### 3.4. Cox Proportional-Hazards Analysis of First CTRCD

In the primary Cox proportional-hazards model, compared with the low-risk group, participants classified as moderate risk had a 2.16-fold higher hazard of first CTRCD (HR 2.16, 95% CI 1.13–4.13; *p* = 0.020), whereas those classified as high/very-high risk had a 3.59-fold higher hazard (HR 3.59, 95% CI 1.42–9.11; *p* = 0.007) (Table 3).

In the adjusted sensitivity model, which included baseline HFA-ICOS risk category, baseline ACEI/ARB and/or beta-blocker therapy, cumulative doxorubicin dose, and left-sided thoracic radiotherapy, the risk gradient remained directionally consistent. Compared with the low-risk group, the HR was 1.66 (95% CI 0.68–4.03; *p* = 0.262) for moderate risk and 2.69 (95% CI 0.86–8.44; *p* = 0.090) for high/very-high risk. Baseline ACEI/ARB and/or beta-blocker therapy (HR 1.70, 95% CI 0.69–4.20; *p* = 0.247), cumulative doxorubicin dose per 50 mg/m^2^ increase (HR 1.10, 95% CI 0.96–1.26; *p* = 0.183), and left-sided thoracic radiotherapy (HR 0.58, 95% CI 0.31–1.09; *p* = 0.093) were not significantly associated with CTRCD (Table 3).

### 3.5. Discrimination of the HFA-ICOS Risk Classification

The ability of the baseline HFA-ICOS classification to discriminate between participants who did and did not experience CTRCD during prospective follow-up was evaluated using ROC analysis. When HFA-ICOS category was treated as an ordinal predictor, the area under the ROC curve (AUC) was 0.630 (bootstrap 95% CI 0.530–0.724), indicating modest discrimination (Figure 3).

Because HFA-ICOS provides categorical risk strata rather than individualized predicted probabilities, conventional probabilistic calibration measures were not calculated.

The ROC curve evaluates the ability of the ordinal baseline HFA-ICOS risk classification to discriminate between participants who did and did not experience documented CTRCD during prospective follow-up. The area under the curve (AUC) was 0.630 (bootstrap 95% CI 0.530–0.724), indicating modest discrimination.

## 4. Discussion

### 4.1. Principal Findings

In this prospective cohort, CTRCD incidence increased stepwise across HFA-ICOS categories: 30.0% in the low-risk, 47.5% in the moderate-risk, and 75.0% in the high/very-high-risk group. CTRCD-free survival differed significantly across risk groups (log-rank χ^2^ = 10.12; *p* = 0.006). In the primary Cox analysis, compared with the low-risk group, the HR was 2.16 (95% CI 1.13–4.13; *p* = 0.020) for moderate risk and 3.59 (95% CI 1.42–9.11; *p* = 0.007) for high/very-high risk. In the exploratory adjusted sensitivity analysis, the stepwise risk gradient remained directionally consistent (HR 1.66 and 2.69, respectively), although the associations were no longer statistically significant. Overall discrimination was modest (AUC 0.630, 95% CI 0.530–0.724), indicating limited individual-level discrimination despite preservation of the ordinal risk gradient.

### 4.2. Interpretation of the Results

The observed ordinal risk gradient, despite modest overall discrimination, is consistent with the intended role of HFA-ICOS as a baseline clinical risk-stratification tool [8,9]. The modest discrimination partly reflects the multifactorial nature of anthracycline-induced cardiotoxicity: the development of CTRCD is influenced by treatment intensity, individual susceptibility, and dynamic myocardial changes during therapy that are not captured by baseline assessment [42,43].

Only 8 of 108 participants (7.4%) were classified as being at high or very high risk, and none had a baseline LVEF < 50%. This may be largely attributable to the relatively young age of the participants (median age, 56 years), which is typical of patients with breast cancer in Kazakhstan [21]. Conditions that confer high cardiovascular risk, including left ventricular systolic dysfunction, are relatively uncommon in middle-aged women. A similar risk distribution has been observed in other cohorts: in the study by Cardinale et al., the very-high-risk category was virtually absent (0 of 2612 and 3 of 819 patients in the development and validation cohorts, respectively) [44], and a low proportion of high-risk patients was also reported in the CARDIOTOX registry [11].

Preserved LVEF does not exclude subclinical LV systolic dysfunction, which may be detected by reduced GLS [31,45]. In the high/very-high-risk group, median LV GLS was −16.7%, approaching the −16% threshold included in the HFA-PEFF criteria for HFpEF [46]. The high CTRCD incidence in the high/very-high-risk group (75.0%) may therefore reflect reduced baseline myocardial functional reserve in some participants that was not apparent from LVEF but was detectable by myocardial deformation parameters [31,45].

No severe asymptomatic or symptomatic CTRCD events were observed in the cohort. Anthracycline cardiotoxicity is regarded as a continuum from subclinical myocardial injury to overt heart failure [47]. When cardiotoxicity is detected and treated early, myocardial dysfunction may be reversible, whereas delayed recognition has been associated with incomplete recovery of LVEF [48,49]. Owing to multiparametric surveillance, 79.1% of CTRCD events were detected during active treatment or by the treatment-completion assessment, after which cardioprotective therapy was initiated or optimized. It may be hypothesized that this contributed to limiting progression of myocardial injury. However, this hypothesis cannot be tested in the absence of a comparison group. The relatively low frequency of severe events may also have been related to the younger age of the participants, preserved baseline LVEF, and moderate cumulative doxorubicin exposure, with a median dose of 237.6 [192.2–315.9] mg/m^2^.

In the exploratory adjusted sensitivity model, the direction of the risk gradient remained consistent, with HRs of 1.66 for moderate risk and 2.69 for high/very-high risk compared with low risk, although the associations were no longer statistically significant. Because all participants in the high/very-high-risk group were receiving an ACEI/ARB at baseline, attenuation of the estimates after adjustment should be interpreted cautiously, particularly given the limited number of events and the substantial overlap between HFA-ICOS category and baseline cardiovascular therapy. A similar pattern was reported by Tini et al.; in their study, patients at moderate and high HFA-ICOS risk more frequently received cardioactive therapy, which may have contributed to the absence of an association between HFA-ICOS risk category and subsequent LV dysfunction [13].

Thus, HFA-ICOS should be considered a tool for initial risk stratification and selection of surveillance intensity rather than a stand-alone predictor of individual CTRCD risk [8,9].

### 4.3. Comparison with Previous Validation Studies

The observed risk gradient was directionally consistent with previous external validation studies, although discrimination and absolute event rates differed appreciably. The largest external validation published to date, the multicenter CARDIOTOX registry (1066 anthracycline-treated patients), reported CTRCD incidences of 2.3%, 6.7%, 19.7%, and 40.0% across the low-, moderate-, high-, and very high-risk categories, with higher discrimination than observed in our cohort (AUC 0.78) [11]. More recently, Suzuki et al. validated HFA-ICOS in the prospective Japanese AIC Registry (339 patients), reporting modest discrimination (ROC AUC 0.599) [12]. Cardinale et al., in a cohort of 2612 patients with baseline LVEF ≥ 50%, reported two-year cardiotoxicity rates of 6.2%, 8.9%, and 11.0% in the low-, moderate-, and high-risk categories, respectively; the AUC was 0.76 in the development cohort and 0.71 in the validation cohort [44].

The higher absolute CTRCD incidence observed in our cohort may be explained in part by differences in outcome definition and surveillance intensity. In these studies, cardiotoxicity was defined predominantly by a decline in LVEF, whereas LV GLS reduction and cardiac biomarker elevation were not included in the outcome definition.

Cardinale et al. noted that the absence of these more sensitive markers may have limited the early detection of myocardial injury and led to underestimation of cardiotoxicity incidence [44].

The ESC multiparametric definition, incorporating LV GLS and cardiac biomarker criteria, combined with protocol-driven surveillance, enables earlier detection of subclinical myocardial injury [45,50]. In our study, such events were documented in 30.0% of women classified as low risk. This narrows the differences in CTRCD incidence between risk categories and reduces the AUC, although the ordinal risk gradient remains preserved.

Our study additionally contributes prospective external validation evidence from a Central Asian population, for which evidence has thus far been limited to preliminary interim reports [36,41], addressing a broader gap noted in the literature regarding the geographic generalizability of HFA-ICOS [51,52].

### 4.4. Clinical Implications

The increasing implementation of cardio-oncology services has highlighted the need for standardized approaches to cardiovascular risk assessment before initiation of potentially cardiotoxic cancer therapy [24]. The HFA-ICOS risk classification was developed to facilitate this process by providing a practical framework for identifying patients who require different intensities of cardiovascular surveillance [8,9]. In the present cohort, the expected ordinal risk gradient was preserved: participants at moderate and high/very-high risk had substantially higher CTRCD incidence and shorter CTRCD-free survival than those at low risk. These findings are consistent with current ESC recommendations for more intensive cardiovascular monitoring in these groups.

At the same time, development of CTRCD in nearly one-third of participants classified as low risk demonstrates that baseline assessment alone is insufficient for individualized prediction. Anthracycline-associated myocardial injury evolves dynamically and is influenced by cumulative exposure, individual susceptibility, and subclinical myocardial changes during treatment [1,37]. Cardiovascular risk assessment should therefore be considered a continuous process rather than a single pretreatment evaluation [48,53]. Baseline HFA-ICOS assessment should determine the initial intensity of surveillance, while subsequent management should be guided by serial echocardiographic findings and cardiac biomarker measurements during treatment [53].

A similar two-stage strategy was recently proposed by Cardinale et al. In this approach, baseline HFA-ICOS stratification is complemented by reassessment after completion of anthracycline therapy using the RE-ACT score, which is based on post-treatment LVEF and cumulative anthracycline dose and improves discriminative performance (AUC 0.87 versus 0.76) [44]. Our findings extend this approach by suggesting that reassessment may benefit from incorporating not only LVEF but also serial changes in LV GLS and cardiac biomarkers, which can detect myocardial injury during treatment. This is consistent with the recent HFA/ESC Council of Cardio-Oncology scientific statement, which emphasizes longitudinal structural, functional, and biomarker surveillance and cautions against reliance on LVEF alone [54].

Prospective validation of HFA-ICOS across different healthcare systems will help clarify the generalizability of the classification and may facilitate implementation of structured cardio-oncology surveillance pathways in routine oncology practice.

### 4.5. Strengths and Limitations

Strengths: Data were collected prospectively, and baseline HFA-ICOS risk classification was assigned before initiation of anticancer therapy and before outcome ascertainment. Consecutive patients were enrolled, and the cohort was homogeneous with respect to the underlying malignancy and anticancer treatment exposure. Cardiovascular surveillance was conducted according to a standardized, predefined longitudinal protocol covering both the active treatment period and subsequent follow-up and incorporating both scheduled and clinically indicated unscheduled assessments. This approach enabled the application of the contemporary multiparametric 2022 ESC definition of cancer therapy-related cardiac dysfunction (CTRCD).

All echocardiographic examinations were performed using the same ultrasound system in a single laboratory by investigators blinded to the HFA-ICOS risk category, with assessment of measurement reproducibility. Baseline risk classification and primary outcome data were complete for all participants. In addition, the use of time-to-event methods allowed assessment not only of the incidence of CTRCD but also of the timing of its development during prospective follow-up.

Limitations: First, this was a single-center study that included only patients with breast cancer, which may limit the generalizability of the findings to other healthcare settings, populations, and malignancies. External validation in larger multicenter cohorts is therefore warranted.

Second, the number of patients classified as high or very high risk was small, and these categories were combined for analysis. Accordingly, estimates for this stratum should be interpreted with caution.

Third, none of the patients in our cohort had a baseline LVEF < 50%. This limits assessment of HFA-ICOS performance in patients with pre-existing LV systolic dysfunction and may also have contributed to the underrepresentation of the highest baseline-risk categories.

Fourth, with 43 primary CTRCD events, the statistical power of the analysis was limited, as reflected by the wide confidence interval around the AUC estimate [55].

Fifth, cardiac biomarkers were measured using point-of-care immunofluorescence assays rather than high-sensitivity laboratory methods. This may have reduced sensitivity for detecting minor biomarker elevations and may therefore have influenced ascertainment of mild CTRCD [56].

Sixth, the primary outcome was dominated by mild, asymptomatic CTRCD (86.0% of events), identified predominantly based on GLS decline. The absence of severe or symptomatic events limits conclusions regarding the performance of HFA-ICOS for more clinically consequential cardiotoxicity and complicates direct comparison with studies using predominantly LVEF-based definitions. When mild CTRCD was detected, cardioprotective therapy was initiated or optimized when clinically indicated. Therefore, the observed course of cardiotoxicity reflects outcomes under active cardiovascular surveillance and treatment, which should be considered when comparing these findings with other cohorts.

Finally, anticancer treatment was unevenly distributed across baseline risk categories. All patients in the high/very-high-risk group received an AC-based regimen without taxane or trastuzumab, whereas these agents were used more frequently in the lower-risk categories. ACE inhibitors/ARBs and beta-blockers were also more commonly used in the higher-risk groups according to clinical indications. These differences in anticancer and cardiovascular treatment may have influenced the observed associations and were partially accounted for in an additional adjusted sensitivity analysis.

## 5. Conclusions

In this prospective external validation, the HFA-ICOS classification demonstrated an increasing observed CTRCD gradient and significant time-to-event separation across baseline risk categories, while overall discrimination remained modest (AUC 0.630). These findings support the role of HFA-ICOS as an initial framework for determining risk-adapted surveillance intensity rather than as a stand-alone predictor of individual cardiotoxicity risk and indicate that baseline classification should be complemented by longitudinal echocardiographic and biomarker monitoring throughout anthracycline-containing therapy. Further evaluations in larger multicenter cohorts, including time-dependent discrimination and assessment of the clinical utility of HFA-ICOS-guided surveillance strategies, would help clarify the broader applicability of these findings.

## Figures and Tables

**Figure 1:**
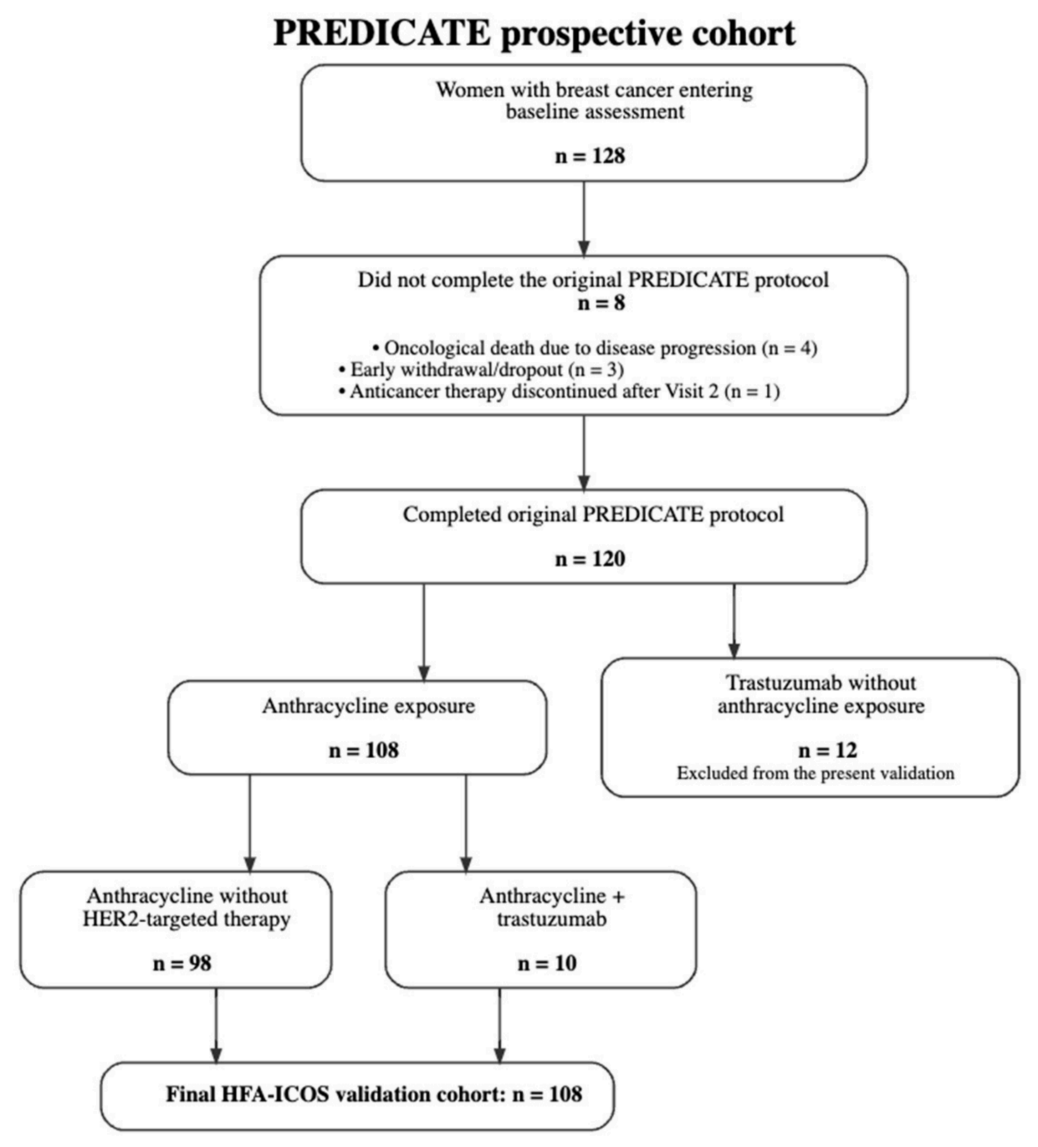
Derivation of the anthracycline-exposed cohort used for external validation of the HFA-ICOS cardiovascular toxicity risk assessment tool within the prospective PREDICATE study.

**Figure 2:**
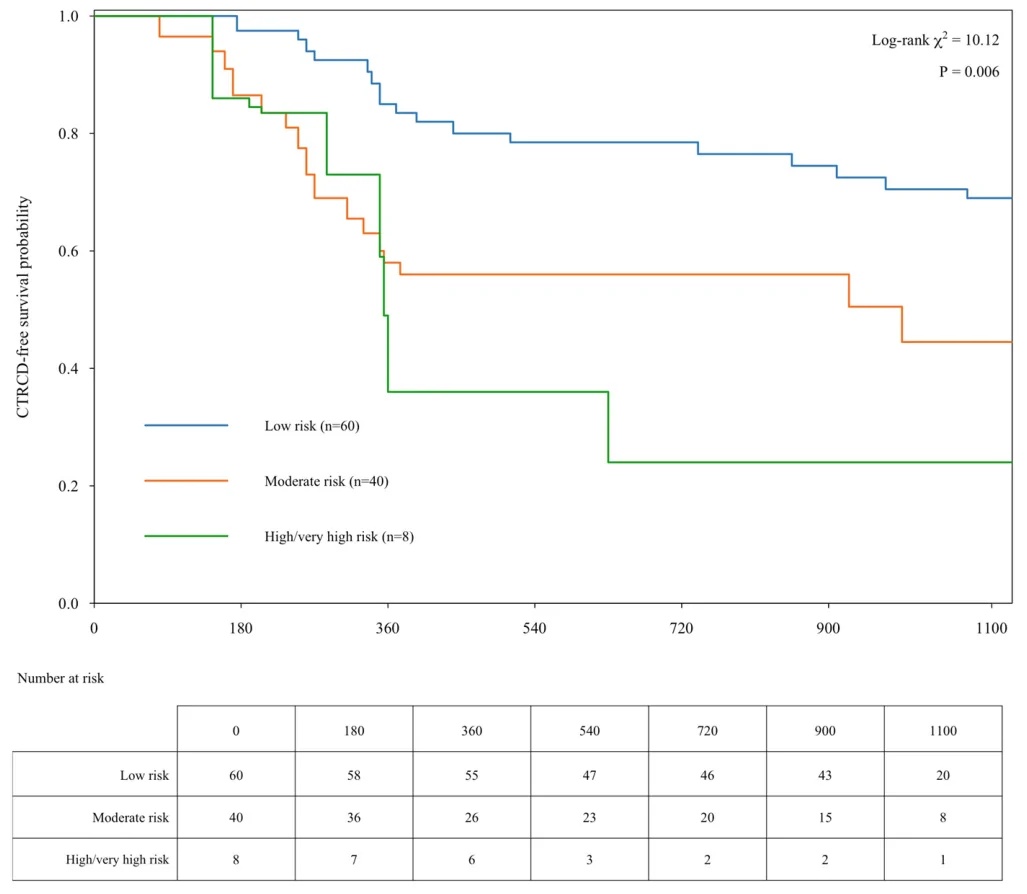
Kaplan–Meier Estimates of CTRCD-Free Survival According to Baseline HFA-ICOS Risk. The survival curves differed significantly across HFA-ICOS risk categories (log-rank χ^2^ = 10.12, *p* = 0.006). Numbers at risk at selected time points are shown below the plot.

**Figure 3:**
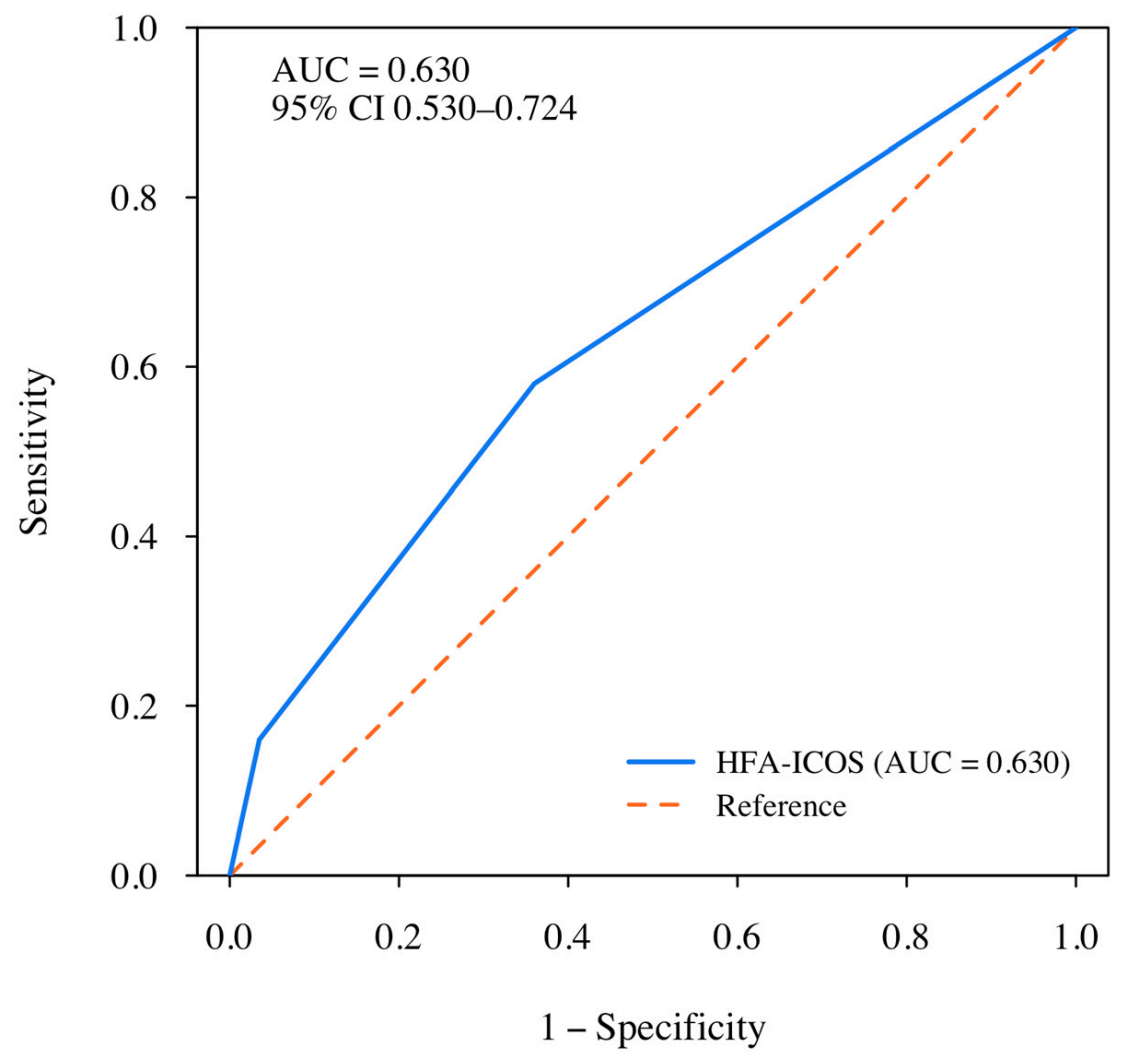
Receiver Operating Characteristic Curve for Discrimination of Subsequent CTRCD by Baseline HFA-ICOS Risk Classification.

**Table 1:** Baseline Clinical, Cardiovascular, Oncological, and Treatment Characteristics of the External Validation Cohort According to HFA-ICOS Risk Category.

Characteristic	Overall (*n* = 108)	Low Risk (*n* = 60)	Moderate Risk (*n* = 40)	High/Very High Risk (*n* = 8)	*p*-Value
Age, years	56.0 [46.8–62.0]	48.0 [41.8–57.2]	60.0 [54.8–65.0]	70.5 [69.0–71.8]	<0.001
Body mass index, kg/m^2^	27.3 [23.3–31.0]	23.9 [22.4–28.5]	31.2 [27.8–36.7]	30.1 [27.8–35.2]	<0.001
Obesity, *n* (%)	36 (33.3)	6 (10.0)	26 (65.0)	4 (50.0)	<0.001
Current/former smoking, *n* (%)	13 (12.0)	7 (11.7)	5 (12.5)	1 (12.5)	1.000
Hypertension, *n* (%)	56 (51.9)	13 (21.7)	35 (87.5)	8 (100.0)	<0.001
Diabetes mellitus, *n* (%)	15 (13.9)	0 (0.0)	12 (30.0)	3 (37.5)	<0.001
Ischemic heart disease, *n* (%)	2 (1.9)	0 (0.0)	0 (0.0)	2 (25.0)	0.005
Chronic kidney disease (eGFR < 60 mL/min/1.73 m^2^), *n* (%)	2 (1.9)	0 (0.0)	1 (2.5)	1 (12.5)	0.060
Systolic blood pressure, mmHg	120 [110–130]	110 [100–120]	130 [120–142]	125 [110–130]	<0.001
Diastolic blood pressure, mmHg	80 [70–80]	70 [70–80]	80 [80–90]	75 [70–80]	<0.001
eGFR, mL/min/1.73 m^2^	91 [80–103]	102 [84–109]	86 [73–98]	82 [75–91]	<0.001
LVEF, %	59.0 [57.0–62.0]	61.0 [58.0–63.0]	58.0 [56.0–60.2]	57.0 [53.0–59.0]	<0.001
LV GLS, %	−18.1 [−19.4 to −16.7]	−18.6 [−19.7 to −17.8]	−17.0 [−18.5 to −16.1]	−16.7 [−19.4 to −15.5]	0.002
cTnI, ng/mL	0.10 [0.10–0.13]	0.10 [0.10–0.10]	0.10 [0.10–0.14]	0.20 [0.10–0.27]	0.020
BNP, pg/mL	49.58 [35.64–68.06]	45.28 [33.48–63.03]	49.84 [38.24–72.68]	84.70 [50.56–116.55]	0.026
Anthracycline without trastuzumab, *n* (%)	98 (90.7)	52 (86.7)	38 (95.0)	8 (100.0)	0.357
Anthracycline + trastuzumab, *n* (%)	10 (9.3)	8 (13.3)	2 (5.0)	0 (0.0)	0.357
Cumulative doxorubicin dose, mg/m^2^	237.6 [192.2–315.9]	248.4 [198.9–320.1]	226.6 [191.2–303.3]	198.9 [166.7–270.9]	0.566
Thoracic radiotherapy, *n* (%)	95 (88.0)	52 (86.7)	35 (87.5)	8 (100.0)	0.810
Left-sided thoracic radiotherapy, *n* (%)	51 (47.2)	23 (38.3)	23 (57.5)	5 (62.5)	0.107
ACEI/ARB, *n* (%)	56 (51.9)	13 (21.7)	35 (87.5)	8 (100.0)	<0.001
Beta-blocker, *n* (%)	2 (1.9)	0 (0.0)	0 (0.0)	2 (25.0)	0.005
Statin, *n* (%)	79 (73.1)	45 (75.0)	28 (70.0)	6 (75.0)	0.887
SGLT2 inhibitor, *n* (%)	0 (0.0)	0 (0.0)	0 (0.0)	0 (0.0)	—

Data are presented as median [interquartile range] or *n* (%). LVEF, left ventricular ejection fraction; LV GLS, left ventricular global longitudinal strain; cTnI, cardiac troponin I; BNP, B-type natriuretic peptide; eGFR, estimated glomerular filtration rate.

**Table 2:** Observed CTRCD Occurrence During Prospective Follow-up According to Baseline HFA-ICOS Risk Category.

HFA-ICOS Risk Category	Participants (*n*)	First CTRCD, *n* (%)	95% CI
Low	60	18 (30.0)	18.8–43.2
Moderate	40	19 (47.5)	31.5–63.9
High/Very High	8	6 (75.0)	34.9–96.8
Overall	108	43 (39.8)	30.5–49.7

**Table 3:** Cox proportional-hazards models for time to first CTRCD according to baseline HFA-ICOS risk.

Variable	Primary Model HR (95% CI)	*p*-Value	Adjusted Sensitivity Model HR (95% CI)	*p*-Value
HFA-ICOS risk category				
Low	Reference	—	Reference	—
Moderate	2.16 (1.13–4.13)	0.020	1.66 (0.68–4.03)	0.262
High/Very High	3.59 (1.42–9.11)	0.007	2.69 (0.86–8.44)	0.090
Baseline ACEI/ARB and/or beta-blocker	—	—	1.70 (0.69–4.20)	0.247
Cumulative doxorubicin dose, per 50 mg/m^2^	—	—	1.10 (0.96–1.26)	0.183
Left-sided thoracic radiotherapy	—	—	0.58 (0.31–1.09)	0.093

HR, hazard ratio; CI, confidence interval; HFA-ICOS, Heart Failure Association–International Cardio-Oncology Society; ACEI, angiotensin-converting enzyme inhibitor; ARB, angiotensin receptor blocker. Low HFA-ICOS risk was used as the reference category.

## Data Availability

The data presented in this study are available on request from the corresponding author. Although patient data were coded anonymously, the informed consent obtained did not extend to open public deposition, and the small, single-center nature of the cohort carries a residual re-identification risk; access is therefore granted upon reasonable request rather than through open deposition.

## Supplementary Materials

The following supporting information can be downloaded at https://www.mdpi.com/article/10.3390/diagnostics16203379/s1. Table S1: Original HFA-ICOS anthracycline risk factors and their operationalization in the PREDICATE external validation cohort; Table S2: Anticancer treatment exposure and cardiovascular therapy initiated after baseline, by HFA-ICOS risk category; Table S3: Timing and severity of first cancer therapy-related cardiac dysfunction (CTRCD) events during active treatment and post-treatment follow-up; Figure S1: Bland–Altman plots of intra- and inter-observer agreement for LVEF and LV GLS.

## Abbreviations

The following abbreviations are used in this manuscript:

AC	doxorubicin–cyclophosphamide
ACEI	angiotensin-converting enzyme inhibitor
AC-T	doxorubicin–cyclophosphamide followed by a taxane
AC-TH	doxorubicin–cyclophosphamide followed by a taxane and trastuzumab
AIC	anthracycline-induced cardiotoxicity
ARB	angiotensin receptor blocker
AUC	area under the curve
BC	breast cancer
BNP	B-type natriuretic peptide
CI	confidence interval
CoV	coefficient of variation
CTRCD	cancer therapy-related cardiac dysfunction
cTnI	cardiac troponin I
eGFR	estimated glomerular filtration rate
ESC	European Society of Cardiology
GLS	global longitudinal strain
HER2	human epidermal growth factor receptor 2
HFA	Heart Failure Association
HFA-ICOS	Heart Failure Association–International Cardio-Oncology Society
HFA-PEFF	Heart Failure Association Pre-test assessment, Echocardiography and natriuretic Peptide, Functional testing, Final etiology
HFpEF	heart failure with preserved ejection fraction
HR	hazard ratio
ICC	intraclass correlation coefficient
ICOS	International Cardio-Oncology Society
IQR	interquartile range
ISRCTN	International Standard Randomised Controlled Trial Number
LV	left ventricular
LVEF	left ventricular ejection fraction
PREDICATE	Prospective Risk Evaluation for the Development of Anthracycline-Induced Cardiotoxicity
ROC	receiver operating characteristic
SPSS	Statistical Package for the Social Sciences
TAC	docetaxel–doxorubicin–cyclophosphamide
