# Supplementary material for: Prospective External Validation of the HFA-ICOS Baseline Cardiovascular Risk Classification in Women with Breast Cancer Receiving Anthracycline-Containing Therapy in a Central Asian Cohort
Source: Diagnostics (Basel). 2026 Oct 17;16(20):3379. doi: 10.3390/diagnostics16203379 (PMC13703341; doi:10.3390/diagnostics16203379)
Supplement: Supplementary file 1 [file diagnostics-16-03379-s001.zip › Supplementary_Figure S1.pdf]

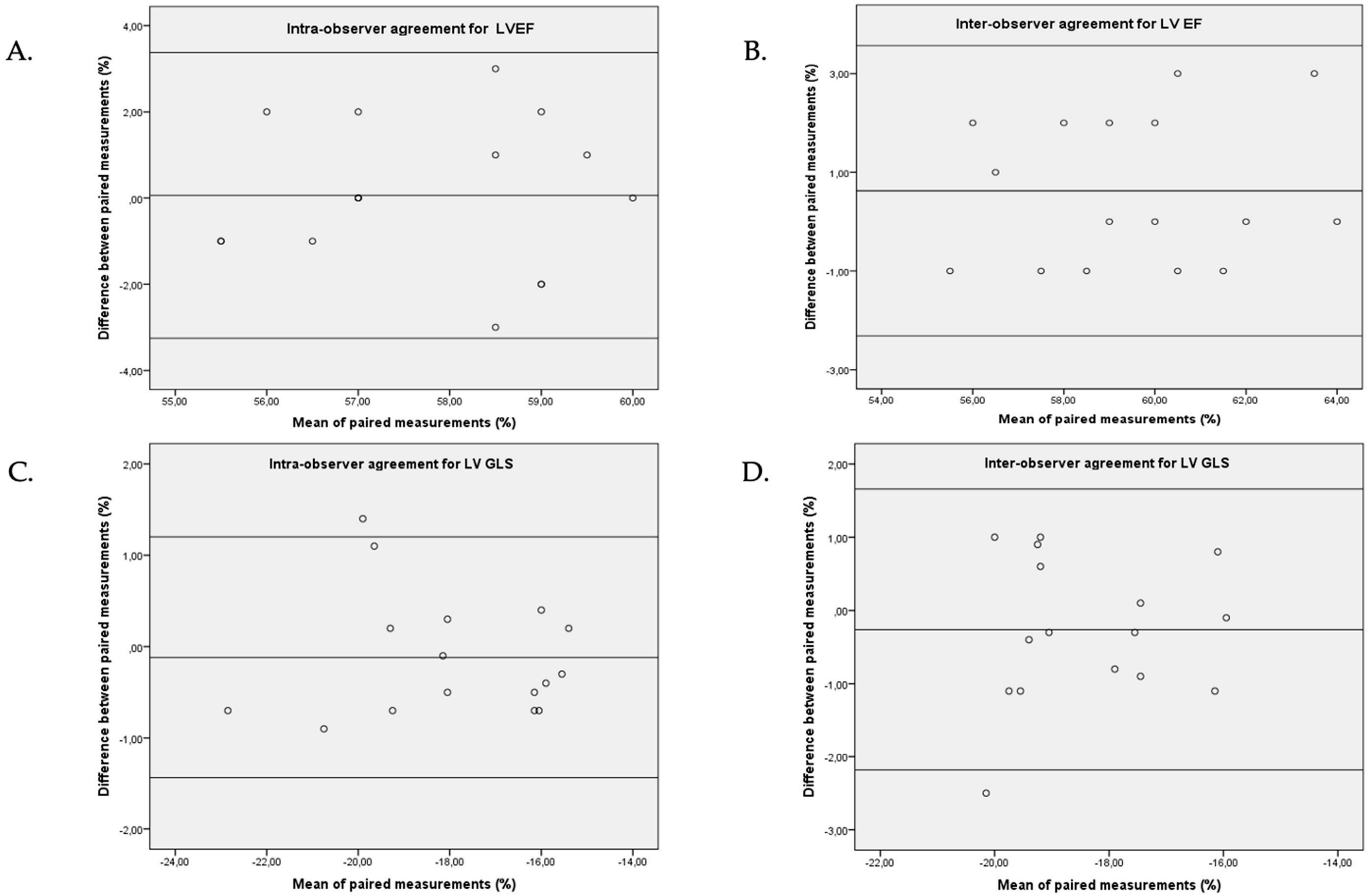

Figure S1. Bland–Altman plots of intra- and inter-observer agreement for LVEF and LV GLS

Measurements were obtained in a subgroup of 16 participants. Panel (A) shows intra-observer agreement for LVEF, panel (B) inter-observer agreement for LVEF, panel (C) intra-observer agreement for LV GLS, and panel (D) inter-observer agreement for LV GLS. The central solid horizontal line indicates the mean bias, and the upper and lower solid lines indicate the 95% limits of agreement. For LVEF, the mean bias was 0.063% with 95% limits of agreement from −3.254% to 3.379% for intra-observer measurements and 0.625% with 95% limits of agreement from −2.315% to 3.565% for inter-observer measurements. For LV GLS, the mean bias was −0.119% with 95% limits of agreement from −1.439% to 1.201% for intra-observer measurements and −0.262% with 95% limits of agreement from −2.183% to 1.658% for inter-observer measurements.

Abbreviations: LV GLS, left ventricular global longitudinal strain; LVEF, left ventricular ejection fraction.
