# Supplementary material for: Prospective External Validation of the HFA-ICOS Baseline Cardiovascular Risk Classification in Women with Breast Cancer Receiving Anthracycline-Containing Therapy in a Central Asian Cohort
Source: Diagnostics (Basel). 2026 Oct 17;16(20):3379. doi: 10.3390/diagnostics16203379 (PMC13703341; doi:10.3390/diagnostics16203379)
Supplement: Supplementary file 1 [file diagnostics-16-03379-s001.zip › Supplementary_Table S1.pdf]

Table S1. **Original HFA-ICOS anthracycline risk factors and their operationalization in the PREDICATE external validation cohort**

The table reports the prespecified components of the original HFA-ICOS baseline cardiovascular risk proforma for anthracycline chemotherapy, together with the operational definitions and source data used in PREDICATE. Only information available before initiation of anticancer therapy was used to derive baseline risk.

HFA-ICOS risk factor	Original weight	Operational definition in PREDICATE	Data source	Missing, n
**Previous cardiovascular disease**				
Heart failure or cardiomyopathy	Very high	Documented diagnosis of heart failure or cardiomyopathy before initiation of the current anticancer treatment.	Baseline medical history, medical records, baseline clinical assessment, transthoracic echocardiography	0
Severe valvular heart disease	High	Documented severe valvular heart disease present before treatment.	Medical history, clinical assessment, and baseline transthoracic echocardiography	0
Myocardial infarction or previous coronary revascularization (PCI or CABG)	High	History of myocardial infarction, percutaneous coronary intervention, or coronary artery bypass grafting before enrollment.	Baseline medical history, medical records, clinical assessment, ECG, and transthoracic echocardiography	0
Stable angina	High	Documented stable angina before initiation of anticancer therapy.	Baseline medical history, medical records, clinical assessment, and ECG	0
Baseline LVEF <50%	High	Baseline left ventricular ejection fraction <50% on transthoracic echocardiography.	Baseline transthoracic echocardiography	0
Borderline LVEF 50–54%	Medium[2]	Baseline left ventricular ejection fraction of 50–54% on transthoracic echocardiography.	Baseline transthoracic echocardiography	0

HFA-ICOS risk factor	Original weight	Operational definition in PREDICATE	Data source	Missing, n
**Cardiac biomarkers**				
Elevated baseline cardiac troponin	Medium[1]	Baseline cardiac troponin above the assay-specific upper reference limit.	Baseline laboratory assessment (cardiac troponin I)	0
Elevated baseline BNP or NT-proBNP	Medium[1]	Baseline natriuretic peptide above the local laboratory upper reference limit; BNP was used in PREDICATE.	Baseline laboratory assessment (BNP)	0
**Demographic and cardiovascular risk factors**				
Age ≥80 years	High	Age at initiation of anticancer therapy ≥80 years.	Baseline clinical assessment	0
Age 65–79 years	Medium[2]	Age at initiation of anticancer therapy 65–79 years.	Baseline clinical assessment	0
Hypertension	Medium[1]	Systolic blood pressure >140 mmHg or diastolic blood pressure >90 mmHg, or current antihypertensive treatment.	Medical history, current antihypertensive therapy, and baseline blood pressure measurement	0
Diabetes mellitus	Medium[1]	Established diabetes mellitus and/or glucose-lowering treatment; classification was based on baseline information available before therapy.	Medical history, current glucose-lowering therapy, and baseline blood glucose measurement	0
Chronic kidney disease	Medium[1]	Estimated glomerular filtration rate <60 mL/min/1.73 m^2 at baseline.	Baseline serum creatinine and eGFR calculated using the CKD-EPI equation	0
**Previous cardiotoxic cancer treatment**				
Previous anthracycline exposure	High	Anthracycline exposure before PREDICATE treatment; current-course exposure was excluded.	Oncology treatment history and medical records	0

HFA-ICOS risk factor	Original weight	Operational definition in PREDICATE	Data source	Missing, n
Prior radiotherapy to the left chest or mediastinum	High	Radiotherapy to the left chest or mediastinum received before the current anticancer treatment course.	Oncology treatment history and medical records	0
Previous non-anthracycline-based chemotherapy	Medium¹	Systemic non-anthracycline chemotherapy received before the current treatment course.	Oncology treatment history and medical records	0
**Lifestyle risk factors**				
Current smoker or significant smoking history	Medium¹	Smoking status and clinically relevant smoking history recorded at baseline.	Baseline medical history	0
Obesity (BMI >30 kg/m²)	Medium¹	Body mass index >30 kg/m² calculated from baseline measured height and weight.	Baseline anthropometric assessment	0

Original HFA-ICOS definitions and weights correspond to the anthracycline chemotherapy proforma [8].

Notes. Medium¹ risk factors contribute 1 point, and Medium² risk factors contribute 2 points. Low risk = no risk factors or one Medium¹ factor; moderate risk = a total of 2–4 medium-risk points; high risk = ≥5 medium-risk points or any high-risk factor; very high-risk = any very-high-risk factor. Elevated cardiac biomarkers were defined relative to the local assay upper reference limit. HFA-ICOS classification was derived exclusively from pretreatment data and was not recalibrated or modified for the present external validation.

All listed HFA-ICOS components were available and applied in the PREDICATE cohort, with no missing baseline predictor data.

In the final anthracycline-exposed validation cohort (n = 108), 60 (55.6%) participants were classified as low risk, 40 (37.0%) as moderate risk, 6 (5.6%) as high and 2 (1.9%) as very high-risk.

Because of the small number of participants in the very high-risk category, the high and very high-risk categories were combined for the principal comparative analyses, resulting in a high/very high-risk stratum of 8 participants (7.4%).

Abbreviations: BMI, body mass index; BNP, B-type natriuretic peptide; CABG, coronary artery bypass grafting; CV, cardiovascular; HFA-ICOS, Heart Failure Association–International Cardio-Oncology Society; LVEF, left ventricular ejection fraction; NT-proBNP, N-terminal pro-B-type natriuretic peptide; PCI, percutaneous coronary intervention; PREDICATE, Prospective Risk Evaluation for the Development of Anthracycline-Induced Cardiotoxicity.
