# Supplementary material for: Prospective External Validation of the HFA-ICOS Baseline Cardiovascular Risk Classification in Women with Breast Cancer Receiving Anthracycline-Containing Therapy in a Central Asian Cohort
Source: Diagnostics (Basel). 2026 Oct 17;16(20):3379. doi: 10.3390/diagnostics16203379 (PMC13703341; doi:10.3390/diagnostics16203379)
Supplement: Supplementary file 1 [file diagnostics-16-03379-s001.zip › Supplementary_Table S2.pdf]

**Table S2. Anticancer treatment exposure and cardiovascular therapy initiated after baseline, by HFA-ICOS risk category**

| Characteristic | Overall (n=108) | Low risk (n=60) | Moderate risk (n=40) | High/Very-high risk (n=8) |
|---|---|---|---|---|
| **Systemic anticancer therapy** | | | | |
| Doxorubicin-containing anthracycline therapy, n (%) | 108 (100.0) | 60 (100.0) | 40 (100.0) | 8 (100.0) |
| Trastuzumab exposure, n (%) | | | | |
| No | 98 (90.7) | 52 (86.7) | 38 (95.0) | 8 (100.0) |
| Yes | 10 (9.3) | 8 (13.3) | 2 (5.0) | 0 (0.0) |
| **Reconstructed chemotherapy regimen** | | | | |
| AC, n (%) | 66 (61.1) | 30 (50.0) | 28 (70.0) | 8 (100.0) |
| AC-T, n (%) | 21 (19.4) | 14 (23.3) | 7 (17.5) | 0 (0.0) |
| AC-TH, n (%) | 10 (9.3) | 8 (13.3) | 2 (5.0) | 0 (0.0) |
| TAC, n (%) | 11 (10.2) | 8 (13.3) | 3 (7.5) | 0 (0.0) |
| Cumulative doxorubicin dose, mg/m$^2$ | 237.6 [192.2-315.9] | 248.4 [198.9-320.1] | 226.6 [191.2-303.3] | 198.9 [166.7-270.9] |
| **Radiotherapy** | | | | |
| Thoracic radiotherapy, n (%) | 95 (88.0) | 52 (86.7) | 35 (87.5) | 8 (100.0) |
| Radiotherapy dose, Gy | 42.5 [30.0-46.0] | 40.0 [28.8-50.0] | 46.0 [30.0-46.0] | 30.0 [25.0-46.0] |
| **Radiotherapy laterality among irradiated participants** | | | | |
| Left-sided, n (% of irradiated) | 51 (53.7) | 23 (44.2) | 23 (65.7) | 5 (62.5) |
| Right-sided, n (% of irradiated) | 42 (44.2) | 28 (53.8) | 11 (31.4) | 3 (37.5) |
| Bilateral, n (% of irradiated) | 2 (2.1) | 1 (1.9) | 1 (2.9) | 0 (0.0) |
| **Surgical treatment** | | | | |
| Breast cancer surgery, n (%) | | | | |
| Yes | 106 (98.1) | 60 (100.0) | 39 (97.5) | 7 (87.5) |
| No | 2 (1.9) | 0 (0.0) | 1 (2.5) | 1 (12.5) |
| **New cardiovascular therapy started post-baseline** | | | | |
| ACE inhibitor/ARB, n (%) | 12 (11.1) | 9 (15.7) | 3 (7.5) | 0 (0.0) |
| Beta-blocker, n (%) | 11 (10.2) | 4 (6.7) | 7 (17.5) | 1 (12.5) |
| Statin, n (%) | 2 (1.9) | 1 (1.7) | 1 (2.5) | 0 (0.0) |
| SGLT2 inhibitor, n (%) | 5 (4.6) | 0 (0.0) | 3 (7.5) | 2 (25.0) |

**Notes.** Data are presented as n (%) or median [interquartile range], unless otherwise indicated.
Chemotherapy regimens were reconstructed from prospectively recorded treatment variables and categorized as AC, AC-T, AC-TH, or TAC for the present analysis.
Post-baseline cardiovascular/cardioprotective therapy was defined as medication newly initiated after the start of anthracycline-containing therapy.

**Abbreviations:** AC, doxorubicin plus cyclophosphamide; AC-T, AC followed by a taxane; AC-TH, AC followed by a taxane and trastuzumab; ACE, angiotensin-converting enzyme; ARB, angiotensin receptor blocker; HFA-ICOS, Heart Failure Association–International Cardio-Oncology Society; SGLT2, sodium–glucose cotransporter-2; TAC, docetaxel, doxorubicin, and cyclophosphamide.
