# Supplementary material for: Prospective External Validation of the HFA-ICOS Baseline Cardiovascular Risk Classification in Women with Breast Cancer Receiving Anthracycline-Containing Therapy in a Central Asian Cohort
Source: Diagnostics (Basel). 2026 Oct 17;16(20):3379. doi: 10.3390/diagnostics16203379 (PMC13703341; doi:10.3390/diagnostics16203379)
Supplement: Supplementary file 1 [file diagnostics-16-03379-s001.zip › Supplementary_Table S3.pdf]

Table S3. Timing and severity of first cancer therapy-related cardiac dysfunction (CTRCD) events during active treatment and post-treatment follow-up

Panel A. Timing and setting of first CTRCD ascertainment

Timing of first CTRCD	Event ascertainment	First CTRCD events, n	% of all first CTRCD events	% of cohort
During active anthracycline-containing treatment or by the treatment-completion assessment	Scheduled serial cardiovascular assessments during treatment and at treatment completion; clinically indicated unscheduled evaluations when CTRCD was suspected	34	79.1	31.5
Post-treatment follow-up	Protocol-defined post-treatment cardiovascular follow-up and clinically indicated unscheduled evaluations	9	20.9	8.3
**Total first CTRCD events**	**—**	**43**	**100.0**	**39.8**

Panel B. Severity of first CTRCD

Severity at first documented CTRCD	First CTRCD events, n	% of all first CTRCD events	% of cohort
Mild asymptomatic CTRCD	37	86.0	34.3
Moderate asymptomatic CTRCD	6	14.0	5.6
Severe asymptomatic CTRCD	0	0.0	0.0
Symptomatic CTRCD	0	0.0	0.0
**Total first CTRCD events**	**43**	**100.0**	**39.8**

Notes. CTRCD timing refers to the first documented fulfillment of the prespecified CTRCD criteria. In the analytical dataset, 34 participants first fulfilled the CTRCD criteria during active treatment or by the treatment-completion assessment, and 9 additional participants first fulfilled the criteria during subsequent post-treatment follow-up, yielding 43 first CTRCD events among 108 participants. Percentages in the fourth column use the 43 first CTRCD events as the denominator; percentages in the fifth column use the full validation cohort (n = 108).
Severity classification: First CTRCD events were classified according to the ESC 2022 cardio-oncology criteria. No severe asymptomatic or symptomatic first CTRCD events were recorded in the validation dataset.
Abbreviations: CTRCD, cancer therapy-related cardiac dysfunction; ESC, European Society of Cardiology.
